# Machine learning to predict high-risk coronary artery disease on CT in the SCOT-HEART trial

**DOI:** 10.1136/openhrt-2025-003162

**Published:** 2025-09-01

**Authors:** Michelle Claire Williams, Alan R M Guimaraes, Muchen Jiang, Jacek Kwieciński, Jonathan R Weir-McCall, Philip D Adamson, Nicholas L Mills, Giles H Roditi, Edwin J R van Beek, Edward Nicol, Daniel S Berman, Piotr J Slomka, Marc R Dweck, David E Newby, Damini Dey

**Affiliations:** 1 British Heart Foundation Centre for Research Excellence, The University of Edinburgh, Edinburgh, Scotland, UK; 2 Department of Interventional Cardiology and Angiology, Institute of Cardiology, Warsaw, Poland; 3 King’s College London, London, UK; 4 Christchurch Heart Institute, University of Otago Christchurch, Christchurch, South Island, New Zealand; 5 University of Glasgow, Glasgow, Glasgow, UK; 6 Royal Brompton and Harefield NHS Foundation Trust, London, London, UK; 7 School of Biomedical Engineering and Imaging Sciences, King’s College London, London, UK; 8 Medicine, Cedars-Sinai Medical Center, Los Angeles, California, USA; 9 Biomedical Imaging Research Institute, Department of Biomedical Sciences, Cedars-Sinai Medical Center, Los Angeles, California, USA

**Keywords:** Computed Tomography Angiography, CORONARY ARTERY DISEASE, Atherosclerosis

## Abstract

**Background:**

Machine learning based on clinical characteristics has the potential to predict coronary CT angiography (CCTA) findings and help guide resource utilisation.

**Methods:**

From the SCOT-HEART (Scottish Computed Tomography of the HEART) trial, data from 1769 patients was used to train and to test machine learning models (XGBoost, 10-fold cross validation, grid search hyperparameter selection). Two models were separately generated to predict the presence of coronary artery disease (CAD) and an increased burden of low-attenuation coronary artery plaque (LAP) using symptoms, demographic and clinical characteristics, electrocardiography and exercise tolerance testing (ETT).

**Results:**

Machine learning predicted the presence of CAD on CCTA (area under the curve (AUC) 0.80, 95% CI 0.74 to 0.85) better than the 10-year cardiovascular risk score alone (AUC 0.75, 95% CI 0.70, 0.81, p=0.004). The most important features in this model were the 10-year cardiovascular risk score, age, sex, total cholesterol and an abnormal ETT. In contrast, the second model used to predict an increased LAP burden performed similarly to the 10-year cardiovascular risk score (AUC 0.75, 95% CI 0.70 to 0.80 vs AUC 0.72, 95% CI 0.66 to 0.77, p=0.08) with the most important features being the 10-year cardiovascular risk score, age, body mass index and total and high-density lipoprotein cholesterol concentrations.

**Conclusion:**

Machine learning models can improve prediction of the presence of CAD on CCTA, over the standard cardiovascular risk score. However, it was not possible to improve the prediction of an increased LAP burden based on clinical factors alone.

**WHAT IS ALREADY KNOWN ON THIS TOPIC**

- Clinical risk scores are widely used to estimate the likelihood of coronary artery disease, but may underestimate or overestimate the prevalence of disease in certain populations.
- Machine learning has shown promise to enhance cardiovascular risk prediction, but its use to identify imaging phenotypes of coronary artery disease is uncertain.

**WHAT THIS STUDY ADDS**

- Machine learning models incorporating routine clinical data improved the prediction of coronary artery disease on coronary CT angiography over the 10-year cardiovascular risk score.
- However, machine learning did not improve the prediction of the presence of high-risk plaque phenotypes such as the increased burden of quantitatively assessed low attenuation plaque.

**HOW THIS STUDY MIGHT AFFECT RESEARCH, PRACTICE OR POLICY**

- Machine learning could be used to optimise waiting lists for patients referred for coronary CT angiography by identifying patients who should be imaged more urgently.

## Introduction

 Coronary CT angiography (CCTA) is now an established technique for the assessment of patients with coronary artery disease. Existing guidelines use a combination of clinical factors to select which patients are referred for CCTA.^1^,^3^ However, it is known that these pretest probability assessments both overestimate and underestimate disease in different populations.^4^ Machine learning (also called artificial intelligence) has shown promise for combining multiparametric clinical information to improve risk prediction.^5^ We hypothesised that it may be possible to use machine learning to better predict which patients are likely to have coronary artery disease or high-risk plaque phenotypes on CCTA.

Machine learning has shown potential to improve risk stratification among patients undergoing a variety of imaging tests.^6 7^ In the CONFIRM (Coronary CT Angiography Evaluation For Clinical Outcomes: An International Multicenter Registry) registry, machine learning improved prediction of all-cause mortality using clinical and imaging findings^6^ and improved prediction of the presence of obstructive coronary artery disease using clinical and calcium score information.^8^ In asymptomatic patients, a machine learning model incorporating multiple clinical and calcium score metrics improved prediction of cardiovascular death compared with the use of these features in isolation.^9^ In the ICONIC (Incident COroNary Syndromes Identified by Computed Tomography) study, machine learning using qualitative and quantitative plaque features improved the identification of the culprit lesions in patients with acute coronary syndromes.^10^ The integration of clinical factors with machine learning may therefore improve the prediction of the presence of coronary artery disease on CT and help optimise patient selection for CCTA.

For patients with stable symptoms undergoing CCTA, the presence of any coronary artery disease and the presence of high-risk plaque phenotypes are both important clinical findings. Identification of coronary artery disease means that patients can be started on medical therapy, while the identification of high-risk plaque phenotypes may identify patients that would benefit from more intensive treatment. Quantitative plaque assessment on CCTA has emerged as a reproducible tool to identify patients with high-risk plaque phenotypes. In particular, the presence of an increased low attenuation plaque burden was associated with an increased risk of fatal and non-fatal myocardial infarction in the Scottish Computed Tomography of the HEART (SCOT-HEART) trial.^11^ The ability to predict the presence of coronary artery disease and high-risk coronary artery disease phenotypes on CCTA would help healthcare professionals to prioritise waiting lists for CCTA.

This study aimed to develop machine learning models using the information available to clinicians at the time of referral for CCTA, to (1) predict the presence of coronary artery disease on CCTA and (2) the presence of an increased low attenuation plaque burden on CCTA. These tools could be used to improve the stratification and prioritisation of patients who are referred for CCTA.

## Methods

### Study design

In the SCOT-HEART trial (ClinicalTrials.gov number, NCT01149590) 4146 patients with stable chest pain were recruited from outpatient clinics from November 2010 through September 2014. Participants were randomised 1:1 to either standard care or standard care plus CT. Inclusion criteria were patients with stable chest pain between 18 and 75 years of age who attended an outpatient cardiology clinic. Of the 2073 patients randomised to CCTA, CCTA was performed and available for subsequent analysis in 1769 patients. These patients formed the study cohort that was used for training and testing in this secondary analysis. The primary results of the trial have previously been published.^12 13^ The funders had no role in the design or conduct of the trial or in the collection, analysis or reporting of data. The steering committee of the SCOT-HEART trial included patient representatives. Where possible, data and code will be shared for checking the reproducibility of the study results on reasonable request to the Chief Investigator under a data sharing agreement.

### Clinical variables

Clinical, imaging and quantitative plaque characteristics were obtained from the SCOT-HEART database. The 10-year cardiovascular risk was calculated using the ASSIGN (Assessing cardiovascular risk using SIGN guidelines) score.^14^ This score is based on the Framingham Risk Score and has been standardised to the Scottish population and well validated. It contains information on age, sex, smoking history, systolic blood pressure, total cholesterol, high density lipoprotein cholesterol, social deprivation index, presence of diabetes mellitus, presence of rheumatoid arthritis and family history of premature cardiovascular disease. Features included in the creation of the machine learning models included demographic characteristics, cardiovascular risk factors, clinical findings, resting ECG findings, exercise ECG results, the 10-year cardiovascular risk score and symptoms obtained from the Seattle Angina Questionnaire (online supplemental Table 1).^15^ These represented the information that was available to clinicians at the time of the initial outpatient chest pain clinic consultation, prior to the patient being randomised and referred for CT. We also compared our machine learning models to the European Society of Cardiology (ESC) pretest probability score which is calculated based on age, gender and symptoms of coronary artery disease as previously described.^1^

### CT imaging and analysis

CCTA was performed using a 64 or 320 detector scanner (Aquilion ONE, Toshiba Medical Systems; Biograph mCT, Siemens; Brilliance 64, Philips Medical Systems). ECG-gated non-contrast CT and ECG-gated contrast-enhanced CCTA was performed with tube current and tube voltage adjusted based on body habitus as previously described.^12 13^ CCTA images were assessed for the presence of coronary artery disease based on a 15-segment model. Coronary artery segments were defined as normal (<10% stenosis), non-obstructive (<70% stenosis or<50% in the left main stem) or obstructive (≥70% stenosis in one or more epicardial vessels or ≥50% in the left main stem). Quantitative plaque analysis was performed using dedicated software in all segments with visually assessed atherosclerotic plaque (Autoplaque, version 2.5, Cedars-Sinai Medical Center). For participants with normal coronary arteries, the plaque volumes and burdens were set to zero. Low-attenuation plaque was classified as plaque with an attenuation of <30 HU (Hounsfield units) as described previously.^11^ Low attenuation plaque burden (%) was calculated as a percentage of the vessel volume of the region assessed. A per patient low attenuation plaque burden above 4% was characterised as an increased low attenuation plaque burden.^11^

For this study, two CCTA-based outcomes were separately assessed. The first outcome was the presence of any coronary artery disease on CCTA, defined as the presence of non-obstructive or obstructive coronary artery disease. The second outcome was the presence of an increased low attenuation plaque burden, defined as a per patient low attenuation plaque burden above 4%.

### Machine learning

Two machine learning models were developed to predict CCTA findings based on clinical information which was available at the time of the initial clinic consultation. The first machine learning model was designed to predict the presence of coronary artery disease on CCTA. The second machine learning model was designed to predict the presence of an increased burden of low attenuation plaque on CCTA.

The machine learning models were built with the XGBoost (eXtreme Gradient Boosting) classification algorithm.^16^ XGBoost uses parallel gradient tree boosting framework to combine a combination of weak decision tree learning algorithms to create a stronger classification model. It employs regularisation techniques to help prevent overfitting and handles missing data by default. Its boosting and gradient-based optimisation help to enable effective learning from complex datasets. Machine learning was performed using R (R Foundation for Statistical Computing, Vienna, Austria, V.4.3.0) and XGBoost (V.1.7.2).

**Figure 1:**
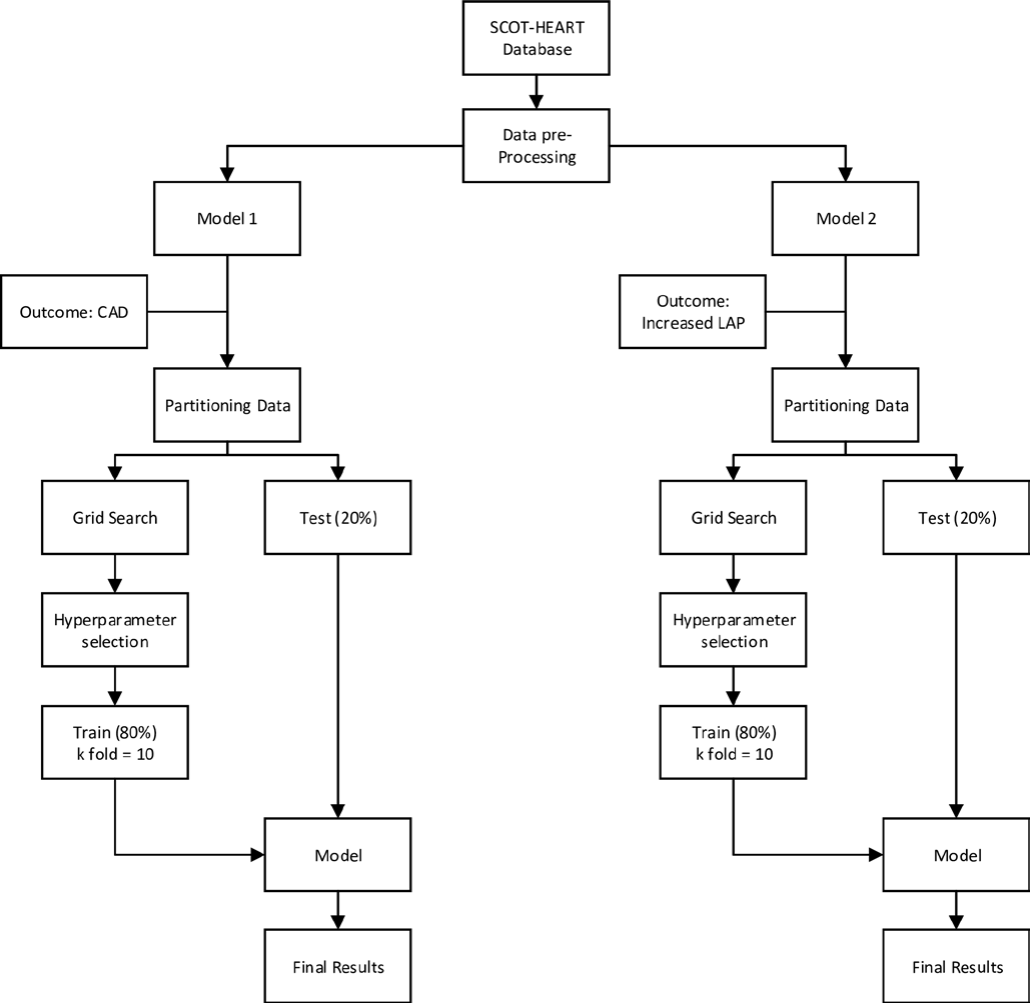
Design of the machine learning models. CAD, coronary artery disease; LAP, low-attenuation coronary artery plaque; SCOT-HEART, Scottish Computed Tomography of the HEART.

The process used to build the machine learning models is shown in figure 1. The dataset was randomly partitioned into two subsets for training and testing, comprising 80% and 20% of the dataset, respectively. Categorical variables were preprocessed using the one-hot encoding method. A grid search was conducted using the training dataset to select optimal hyperparameters for each model (online supplemental table 1 and 2). Model training was performed with 10-fold cross-validation. The predictive abilities of the resulting models were tested on the separate hold-out testing datasets.

### Understanding the machine learning models

To understand the influence of each of the variables included in the machine learning models, we assessed the feature importance scores from the XGBoost models. Feature importance is a relative measure of how much each feature contributes to improving the performance of the XGBoost model. In addition, individual waterfall plots were constructed for randomly selected patients with and without coronary artery disease on CCTA and with and without an increased burden of low attenuation plaque on CCTA. These demonstrate the contribution of variables to the prediction of CCTA findings from the XGBoost model in individual patients.

### Statistical analysis

Statistical analysis was performed with R (R Foundation for Statistical Computing, Vienna, Austria; V.4.3.0). Normally distributed data are displayed as mean and SD and non-normally distributed data are displayed as median and IQR. Statistical significance was assessed with Pearson’s χ^2^ test, Fisher’s exact test, Student’s t-test or Mann-Whitney U test, as appropriate. Results were considered statistically significant if a two-sided p value was<0.05. Receiver operator curves (ROC) were constructed and area under the curve (AUC) is presented along with the 95% CIs. ROC curves were compared with the DeLong test.

## Results

### Study population

Of the 1769 patients who underwent CCTA which were available for analysis, 56% (n=977) were male, the mean age was 58±9.5 years and the mean 10-year cardiovascular risk score was 16% (IQR 10%–23%) (table 1). The dataset was randomly divided into 1416 (80%) patients who were used for training and 353 (20%) patients who were used for hold-out testing. The demographic characteristics of the training and testing subsets of the cohort were similar (table 1).

**Table 1:** Baseline characteristics of the training and testing subsets of the dataset

	Overall	Training	Testing	P value
Number	1769	1416	353	
Male sex (%)	997 (56)	808 (57)	189 (54)	0.257
Age (years)	58±9.5	55±9.5	58±9.1	0.723
Body mass index (kg/m^2^)	30±5.5	30±5.5	30±5.7	0.778
Diabetes mellitus (%)	196 (11)	155 (11)	41 (12)	0.792
Previous history of CAD (%)	178 (10)	144 (10)	34 (9.6)	0.84
Atrial fibrillation (%)	34 (1.9)	27 (1.9)	7 (2.0)	1
Current smoker (%)	330 (19)	259 (18)	71 (20)	0.463
Ex-smoker (%)	593 (34)	472 (33)	121 (34)	0.759
Non-smoker (%)	845 (48)	685 (48)	160 (46)	0.356
Hypertension (%)	608 (35)	490 (35)	118 (34)	0.736
Total cholesterol (mg/dL)	192±73	192±73	195±69	0.384
High density lipoprotein (mg/dL)	37±27	37±26	37±30	0.953
Family history of CAD (%)	765 (44)	620 (44)	145 (41)	0.389
Previous history of CVD (%)	79 (4.5)	69 (4.9)	10 (2.8)	0.125
Previous history of PVD (%)	31 (1.8)	26 (1.8)	5 (1.4)	0.755
Normal rest electrocardiogram (%)	1499 (86)	1192 (85)	307 (88)	0.207
Exercise tolerance test abnormal (%)	235 (17)	189 (17)	46 (16)	0.901
Exercise tolerance test inconclusive (%)	255 (18)	201 (18)	54 (19)	0.693
Exercise tolerance test normal (%)	919 (65)	736 (65)	183 (65)	0.88
Atypical angina (%)	432 (24)	348 (25)	84 (24)	0.813
Non-anginal chest pain (%)	683 (39)	536 (38)	147 (42)	0.212
Typical angina (%)	654 (37)	532 (38)	122 (35)	0.324
Hyperlipidaemia (%)	1078 (61)	859 (61)	219 (62)	0.68
10-year cardiovascular risk score (%)	16 (10–23)	15 (10–23)	16 (9–23)	0.769
Any coronary artery disease (%)	1123 (64)	899 (64)	224 (64)	1
Increased low attenuation plaque burden* (%)	907 (51)	725 (51)	182 (52)	0.952

Number (%). Mean±SD.

* Increased low attenuation plaque burden defined as >4%.

CAD, coronary artery disease; CVD, cerebrovascular disease; PVD, peripheral vascular disease.

### Identification of coronary artery disease on CCTA

**Figure 2:**
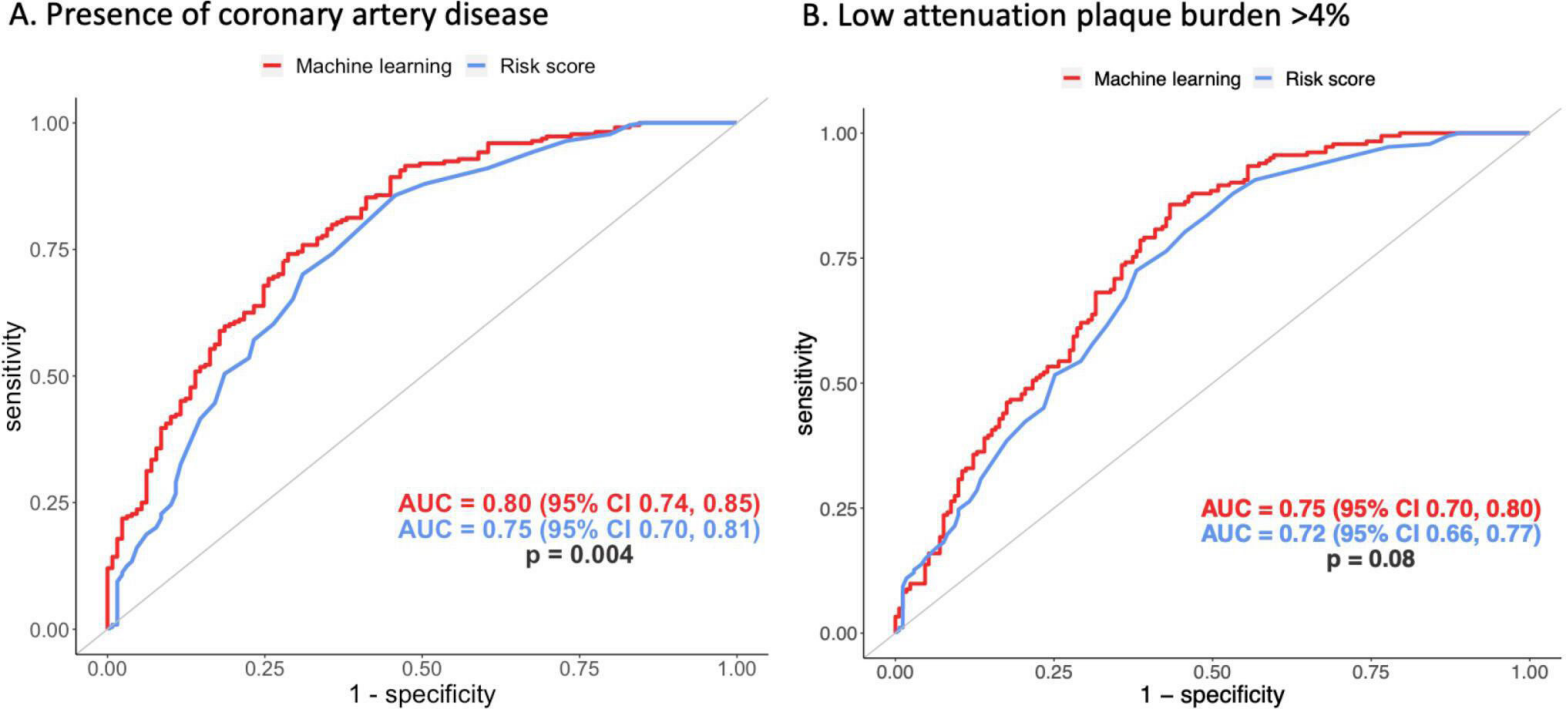
Receiver operator curves showing the performance of the two machine learning models (red) and the 10-year cardiovascular risk score (blue) to predict the presence of (**A**) any coronary artery disease on coronary CT angiography and (**B**) an increased burden of low attenuation plaque (>4%). AUC, area under the curve.

Coronary artery disease (>10% stenosis) was identified on 64% of CCTA (n=1123), including 899 patients (64%) in the training dataset and 224 patients (64%) in the testing dataset. In the hold-out testing dataset, the 10-year cardiovascular risk score could predict the presence of coronary artery disease on CCTA with an AUC of 0.75 (95% CI 0.70 to 0.81). The machine learning model showed an improved ability to predict the presence of coronary artery disease on CCTA with an AUC of 0.80 (95% CI 0.74 to 0.85) and p value for ROC curve comparison of 0.004 (figure 2, table 2). In addition, the machine learning model showed improved identification of coronary artery disease compared with ESC pretest probability model (AUC 0.73, 95% CI 0.68 to 0.79, p<0.001, online supplemental figure 1).

**Table 2:** Contingency table for the detection of coronary artery disease (CAD) and low attenuation plaque (LAP)

	True positive	True negative	False positive	False negative	Sensitivity	Specificity	PPV	NPV	AUC
Model 1 CAD detection	163	93	36	61	72%	72%	60%	82%	0.80
Model 2 LAP detection	131	110	61	51	64%	72%	68%	86%	0.75

AUC, area under the curve; NPV, negative predictive value; PPV, positive predictive value.

**Figure 3:**
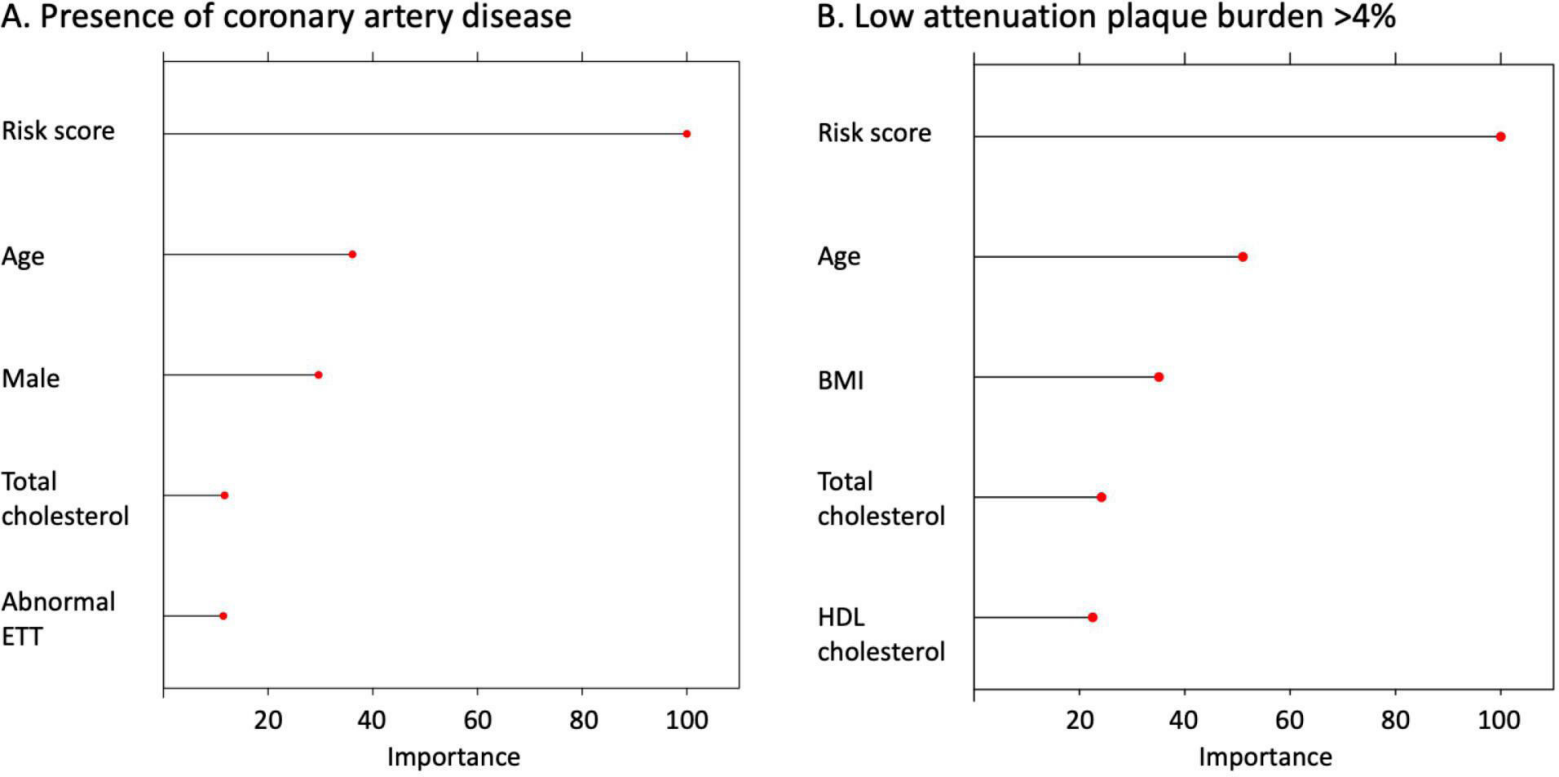
Feature importance plots for the top five features included in the models for identifying (**A**) the presence of any coronary artery disease on coronary CT angiography and (**B**) the presence of an increased burden of low attenuation plaque (>4%). BMI, body mass index; ETT, exercise tolerance test; HDL, high-density lipoprotein.

**Figure 4:**
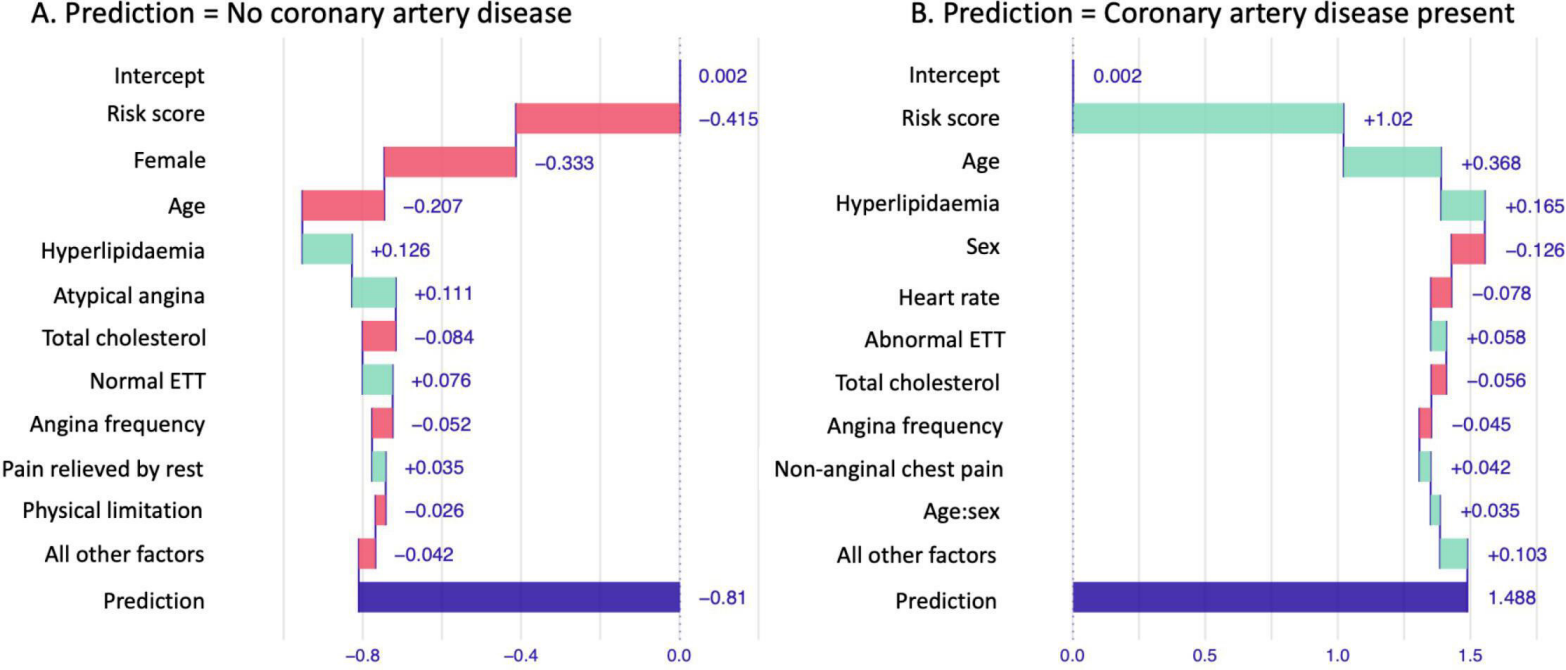
Waterfall plots showing a visual representation of the features used by the machine learning model for the prediction of the presence of any coronary artery disease on coronary CT angiography in two separate patients. The first patient (**A**) was a female aged 54 years with a 10-year cardiovascular risk score of 16% with no coronary artery disease on CCTA. The second patient (**B**) was male aged 67 years with a 10-year cardiovascular risk score of 14% and coronary artery disease on CCTA. CCTA, coronary CT angiography; ETT, exercise tolerance testing.

Feature importance assessment showed that the most important characteristics in the machine learning model for the identification of coronary artery disease was the 10-year cardiovascular risk score, age, sex, total cholesterol and the presence of an abnormal exercise tolerance test (figure 3). The features used by the model for prediction in two separate patients are shown in figure 4, showing the differences between patients with and without coronary artery disease on CCTA.

### Identification of an increased low attenuation plaque burden on CCTA

An increased burden of low attenuation plaque was identified in 51% of patients (n=907), including 725 (51%) in the training dataset and 182 (52%) in the testing dataset. In the hold-out testing dataset, the machine learning model had a similar ability to predict the presence of an increased burden of low attenuation plaque compared with the 10-year cardiovascular risk score (AUC 0.75, 95% CI 0.70 to 0.80 vs AUC 0.72, 95% CI 0.66 to 0.77, p=0.08 for comparison, figure 2, table 2). The machine learning model predicted the presence of an increased low attenuation plaque burden slightly better than the ESC pretest probability score (AUC 0.69, 95% CI 0.64 to 0.74, p=0.005, online supplemental figure 1).

**Figure 5:**
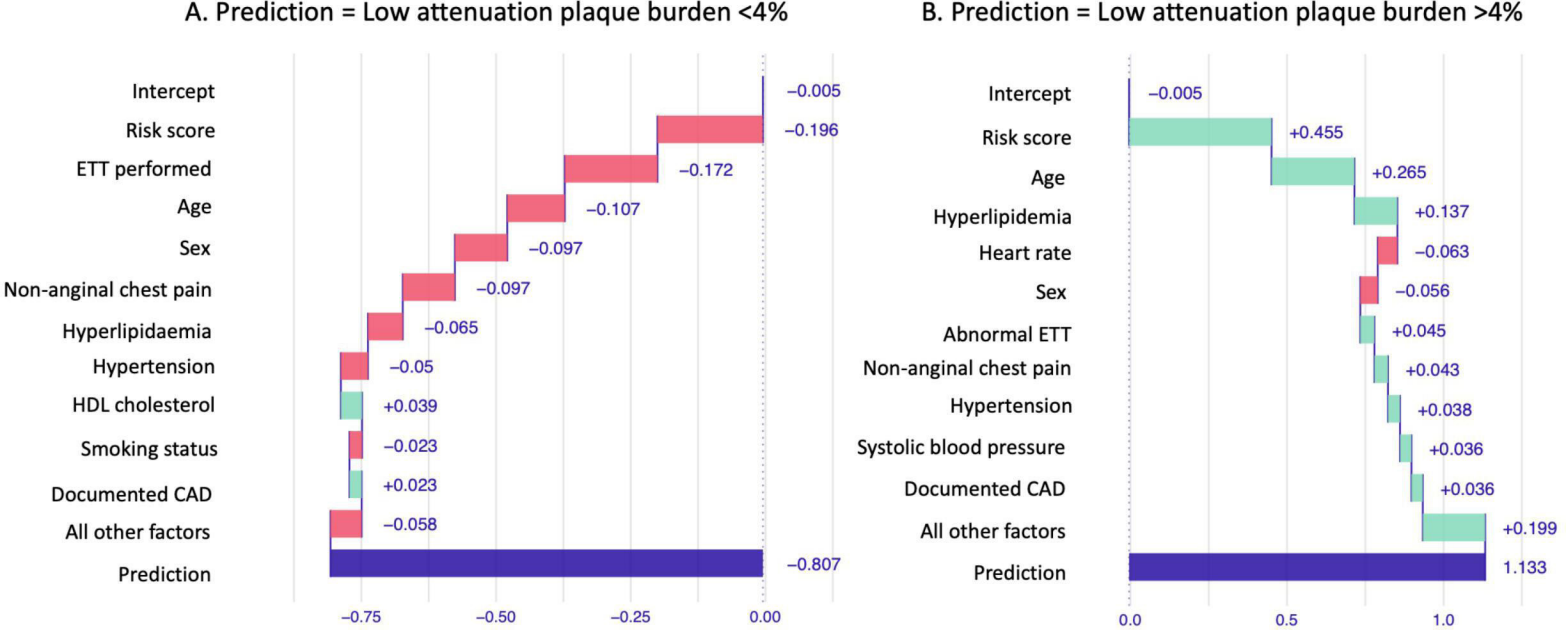
Waterfall plots showing a visual representation of the features used by the machine learning model for the prediction of the presence of an increased burden of low attenuation plaque on coronary CT angiography in two patients. The first patient (**A**) was a female aged 70 years with a 10-year cardiovascular risk score of 35% with a low attenuation plaque <4% on CCTA. The second patient (**B**) was a male aged 67 years with a 10-year cardiovascular risk score of 14% and a low attenuation plaque burden >4% on CCTA. CAD, coronary artery disease; CCTA, coronary CT angiography; ETT, exercise tolerance testing; HDL, high density lipoprotein.

Feature importance assessment showed that the most important characteristics in the machine learning model for the identification of an increased burden of low attenuation plaque were the 10-year cardiovascular risk score, age, body mass index, total cholesterol and high-density lipoprotein cholesterol. The features used by the model for prediction in two separate patients are shown in figure 5, showing the differences between a patient with and a patient without a low attenuation plaque burden above 4% on CCTA.

## Discussion

In this study, we have shown that supervised machine learning can be used to identify patients who are likely to have coronary artery disease on CCTA based on clinical features and that this provides additive predictive ability over and above the cardiovascular risk score. However, machine learning with these features did not improve the identification of patients with a high burden of low attenuation plaque on CCTA compared with the risk score. This shows that for patients with suspected angina due to coronary artery disease, the baseline demographics are not enough to identify patients with high-risk plaque characteristics on CCTA. The information provided by these models could therefore be used to prioritise waiting lists for CCTA.

Recently, machine learning has shown great potential in cardiovascular imaging, with wide-ranging techniques being developed which include improved image acquisition, image annotation, diagnosis and risk prediction.^5^ For risk prediction, machine learning models have been developed to identify obstructive coronary artery disease on CT, culprit coronary artery plaques and to bring together clinical and imaging data to improve risk stratification.^68^,^10^ Previous studies have focused on the identification of obstructive coronary artery disease on CT to try and identify patients who have more severe disease and may benefit from revascularisation. However, in our study, we focused on the identification of patients who had any coronary artery disease and would benefit from preventative medical therapies. In our study, we have developed a machine learning model that can identify patients who are likely to have coronary artery disease on CT with improved accuracy over and above the 10-year cardiovascular risk score or ESC pretest probability score. Importantly, this model used the information that was available at the time of the initial clinic consultation, including current symptoms, medical history, examination, ECG and exercise tolerance test findings. This information could be used to prioritise patients who would benefit from more urgent investigation and highlight patients who should be started on preventative therapy while they wait for the results of their CCTA.

Quantitative assessment of coronary artery plaque can improve patient assessment by highlighting both the amount and characteristics of the coronary artery plaque. The amount of total plaque and the low attenuation plaque burden, in particular, have been shown to be markers of particularly high-risk coronary artery disease phenotypes.^11 17^ These patients may benefit from more intensive medical therapy. In our study, the machine learning model was as good as the standard 10-year cardiovascular risk score (ASSIGN) at predicting which patients would have an increased low attenuation plaque burden but did not provide additive predictive value. Our machine learning model for the prediction of LAP was slightly better than the ESC pretest probability score, but the ESC pretest probability score was not optimised for the Scottish population. This finding underlines the importance of performing imaging for patients with stable chest pain to identify those at greatest risk of cardiovascular events. New metrics from CCTA may be useful to further improve risk stratification, such as radiomic characteristics,^18^ and further research is required in this area.

Importantly, we used two different techniques to understand the factors that the machine learning models were using to perform the predictions. So-called ‘explainable AI’ is an important part of machine learning model development as we seek to understand the model performance and gain trust in its performance.^19^ We assessed feature importance plots to assess the most important features used across all predictions in the testing dataset and additionally used waterfall plots to look at the factors which impacted predictions in individual patients. Interestingly, different parameters were used by the two models developed in our study. The cardiovascular risk score, age and total cholesterol were in the top five features included in both the model to predict the presence of coronary artery disease and the presence of an increased burden of low attenuation plaque. However, for the model predicting the presence of coronary artery disease, the top five features also included sex and an abnormal exercise tolerance test. In contrast, for the model predicting an increased burden of low attenuation plaque, the body mass index and high-density lipoprotein cholesterol were among the top five most important features. This highlights important risk factors in the development of different types of coronary artery disease.

This study has some limitations that should be acknowledged. First, there is not a currently suitable dataset for external testing of these models and developing such a dataset is important prior to their clinical application. Second, only the routinely collected clinical characteristics were used for model development and additional factors may help improve the accuracy of the model in future. Third, these models were developed in patients with stable chest pain and may not be applicable to other clinical scenarios such as asymptomatic patients or emergency department patients. In addition, in clinical practice, there may be missing variables which prevent the full use of machine learning models. We used the ASSIGN cardiovascular risk score and the ESC pretest probability score as comparators, which may not be applicable to all populations. In addition, these scores were not designed for the prediction of coronary artery disease. Other non-machine learning methods could also be used to develop new risk scores for the presence of disease on CT. Fourth, the number of patients used for training and testing in this manuscript is small. Finally, we used three different CT scanners to image patients in the SCOT-HEART trial, which may have impacted the quantitative plaque analysis.

In this study, we have shown that machine learning can be used to improve prediction of the presence of coronary artery disease on CCTA, but not the presence of an increased burden of low attenuation plaque. This could be used to help prioritise patients for more urgent imaging and underlines the importance of performing CCTA for accurate diagnosis.

## Supplementary material

10.1136/openhrt-2025-003162 online supplemental file 1

## Data Availability

Data are available upon reasonable request.
