# Supplementary material for: Machine learning to predict high-risk coronary artery disease on CT in the SCOT-HEART trial
Source: Open Heart. 2025 Sep 1;12(2):e003162. doi: 10.1136/openhrt-2025-003162 (PMC12406813; doi:10.1136/openhrt-2025-003162)
Supplement: online supplemental file 1 [file openhrt-12-2-s001.docx]

**Supplementary information**

Supplementary Table 1: Features included in the machine learning models.

| **Type of parameter** | **Parameter** |
| --- | --- |
| Demographic characteristics | Age |
|  | Age >65 years |
|  | Sex |
|  | Body mass index |
|  | Body mass index >30 kg/m^2^ |
|  | Height |
|  | Weight |
| Cardiovascular risk factors | Diabetes mellitus |
|  | Type 1 diabetes mellitus |
|  | Type 2 diabetes mellitus |
|  | Type 1 diabetes mellitus – diet controlled |
|  | Type 1 diabetes mellitus – oral hypoglycaemics |
|  | Type 1 diabetes mellitus – on insulin |
|  | Type 2 diabetes mellitus – diet controlled |
|  | Type 2 diabetes mellitus – oral hypoglycaemics |
|  | Type 2 diabetes mellitus – on insulin |
|  | Prior history of coronary heart disease |
|  | Atrial fibrillation |
|  | Smoking habit |
|  | Cigarettes per day |
|  | Hypertension |
|  | Total cholesterol |
|  | HDL cholesterol |
|  | Family history of coronary artery disease |
|  | Previous history of cerebrovascular disease |
|  | Previous history of peripheral vascular disease |
|  | Hyperlipidaemia |
|  | 10 year cardiovascular risk score (ASSIGN score) |
| Examination | Systolic blood pressure |
|  | Diastolic blood pressure |
|  | Vascular bruits |
|  | Ejection systolic murmur |
|  | Heart rate |
| Symptoms | History of constricting chest pain |
|  | History of chest pain precipitated by exercise |
|  | History of chest pain relieved by rest |
|  | Typical, atypical or non-anginal chest pain |
|  | Seattle angina questionnaire - Physical limitation |
|  | Seattle angina questionnaire – angina stability |
|  | Seattle angina questionnaire – angina frequency |
|  | Quality of life |
| Resting electrocardiogram | Normal |
|  | ST segment deviation |
|  | Inferior ST segment deviation |
|  | Anterior ST segment deviation |
|  | Lateral ST segment deviation |
|  | ST segment elevation |
|  | ST segment depression |
|  | T wave inversion |
|  | Inferior T wave inversion |
|  | Anterior T wave inversion |
|  | Lateral T wave inversion |
|  | Pathological Q waves |
|  | Inferior pathological Q waves |
|  | Anterior pathological Q waves |
|  | Lateral pathological Q waves |
|  | Bundle branch block |
|  | Left bundle branch block |
|  | Right Left bundle branch block |
|  | Left ventricular hypertrophy |
| Exercise tolerance test | Exercise electrocardiogram performed |
|  | Exercise electrocardiogram outcome |

ASSIGN, Assessing cardiovascular risk using SIGN guidelines.

Supplementary Table 2: Optimal hyperparameters for the model to identify the presence of any coronary artery disease on coronary computed tomography angiography.

| **Hyperparameter** | **Value** |
| --- | --- |
| nrounds | 1000 |
| Max depth | 2 |
| eta | 0.004 |
| gamma | 1 |
| colsample_bytree | 1 |
| min_child_weight | 1 |
| subsample | 0.6 |

Supplementary Table 3: Optimal hyperparameters for the model designed to identify an increased burden of low attenuation plaque on coronary computed tomography angiography.

| **Hyperparameter** | **Value** |
| --- | --- |
| nrounds | 500 |
| Max depth | 4 |
| eta | 0.005 |
| gamma | 0 |
| colsample_bytree | 0.3 |
| min_child_weight | 1 |
| subsample | 0.5 |

Supplementary Figure 1. Receiver operator curves showing the performance of the two machine learning models (red) and the ESC pre-test probability score (blue) to predict the presence of (A) any coronary artery disease on coronary computed tomography angiography and (B) an increased burden of low attenuation plaque (>4%).
